# A Comparison of Evoked and Non-evoked Functional Networks

**DOI:** 10.1007/s10548-018-0692-1

**Published:** 2018-12-06

**Authors:** Jurgen Hebbink, Dorien van Blooijs, Geertjan Huiskamp, Frans S. S. Leijten, Stephan A. van Gils, Hil G. E. Meijer

**Affiliations:** 1 Department of Neurology and Neurosurgery, Brain Center Rudolf Magnus, University Medical Centre Utrecht, Heidelberglaan 100, 3584 CX Utrecht, The Netherlands; 2 Department of Applied Mathematics, MIRA Institute for Biomedical Engineering and Technical Medicine, University of Twente, Drienerlolaan 5, 7500 AE Enschede, The Netherlands

**Keywords:** Brain networks, Functional connectivity, Single pulse electrical stimulation, Cortico-cortical evoked potentials, Electrocorticography

## Abstract

The growing interest in brain networks to study the brain’s function in cognition and diseases has produced an increase in methods to extract these networks. Typically, each method yields a different network. Therefore, one may ask what the resulting networks represent. To address this issue we consider electrocorticography (ECoG) data where we compare three methods. We derive networks from on-going ECoG data using two traditional methods: cross-correlation (CC) and Granger causality (GC). Next, connectivity is probed actively using single pulse electrical stimulation (SPES). We compare the overlap in connectivity between these three methods as well as their ability to reveal well-known anatomical connections in the language circuit. We find that strong connections in the CC network form more or less a subset of the SPES network. GC and SPES are related more weakly, although GC connections coincide more frequently with SPES connections compared to non-existing SPES connections. Connectivity between the two major hubs in the language circuit, Broca’s and Wernicke’s area, is only found in SPES networks. Our results are of interest for the use of patient-specific networks obtained from ECoG. In epilepsy research, such networks form the basis for methods that predict the effect of epilepsy surgery. For this application SPES networks are interesting as they disclose more physiological connections compared to CC and GC networks.

**Electronic supplementary material:**

The online version of this article (10.1007/s10548-018-0692-1) contains supplementary material, which is available to authorized users.

## Introduction

Brain networks are increasingly being studied as they may aid in understanding the brain’s function in cognition (Mill et al. 2017; Park and Friston 2013) and diseases, such as Alzheimer’s disease (Tijms et al. 2013), epilepsy (Bartolomei et al. 2017; Engel et al. 2013; van Mierlo et al. 2014) and schizophrenia (van den Heuvel and Fornito 2014). A recent development is to incorporate brain networks in computational models for epilepsy surgery (Goodfellow et al. 2016; Khambhati et al. 2016; Sinha et al. 2017). Networks consist of nodes, representing neuronal populations, which are connected via edges. Based on the interpretation of the edges networks can be categorized as structural, functional or effective (Rubinov and Sporns 2010). The concept of structural networks is the most intuitive; edges simply describe anatomical connections between neuronal populations. The presence of such an anatomical connection, however, does not indicate how intensively it is used in communication between the neuronal populations. Functional and effective connectivity methods try to assess this point. In functional connectivity edges describe statistical dependencies among time series of neuronal activity (Wang et al. 2014), while effective connectivity is defined as the influence one neuronal system exerts over another (Friston 2011).

Methods for functional connectivity use simultaneously recorded time series which can be acquired via a large variety of imaging modalities, e.g. electroencephalography (EEG). Connectivity is then calculated from the band-filtered time series or their envelopes (Keller et al. 2013) using methods like cross-correlation (CC) (Kramer et al. 2009), Granger causality (GC) (Bressler and Seth 2011) and mutual information (Pluim et al. 2003). Almost all these methods have a mathematical foundation that makes assumptions about the processes underlying the observations (Wang et al. 2014). In practice, most of these assumptions only hold to some extent and one may wonder how this influences the obtained connectivity

Interventional approaches, in contrast, actively perturb activity at some location using electric or magnetic pulses in order to observe neural responses at other sites (Keller et al. 2014) and hence they infer connectivity in a more direct way than non-interventional approaches. Networks derived in this way are called evoked effective networks (Keller et al. 2014). Pre-surgical evaluation of refractory focal epilepsy patients offers a unique setting to apply this approach in an invasive setting. In these patients electrocorticography (ECoG), i.e. an invasive form of EEG, may be recorded using an electrode grid placed directly on the cortex. Single pulse electrical stimulation (SPES) (Mouthaan et al. 2016) applies brief electric pulses to adjacent pairs of electrodes of this grid. These pulses have a typical duration of 0.1–3 ms and a strength of 2–12 mA (Donos et al. 2016a) and evoke responses, called cortico-cortical evoked potentials (CCEP), at the non-stimulated electrodes. Commonly, two types of responses are distinguished in SPES literature, i.e. early responses (ERs) and delayed responses (DRs) (Valentín et al. 2002). ERs occur within 100 ms. It is widely thought that they represent direct cortico-cortical propagation (Lacruz et al. 2007; Matsumoto et al. 2017; Entz et al. 2014). For completeness, we mention that DRs are typical for epileptogenic tissue (Valentín et al. 2002; van’t Klooster et al. 2011).

SPES offers a more direct approach to infer networks than functional connectivity. Functional connectivity, however, can be applied to recordings of on-going ECoG activity as well as to non-invasive imaging methods like scalp EEG making it more accessible than SPES. While relations between structural and evoked effective networks have been studied (Conner et al. 2011; Donos et al. 2016b; Parker et al. 2018), it is not known what functional networks constructed using on-going ECoG have in common with SPES-evoked connectivity. Do they find the same connections? Do they reveal well-known anatomical connections?

To answer these questions we will construct networks for six patients using three different methods. One is the SPES network while the other two are CC and GC networks both derived from on-going inter-ictal ECoG. We will compare the connections between these networks and investigate to what extent those networks can unravel connectivity in an established functional network, i.e. the language circuit containing Broca’s and Wernicke’s area.

## Materials and Methods

### Data Selection and Pre-processing

We use ECoG data, recorded with grid electrodes, of six patients with focal epilepsy who underwent long-term ECoG monitoring prior to surgery at the University Medical Centre Utrecht. Data are retrospectively studied and handled coded and anonymously according to the guidelines of the institutional ethical committee. Patient characteristics are provided in Table 1. For each patient, SPES has been performed as part of clinical routine. ECoG data has been recorded using a common reference montage with respect to an extracranial reference electrode. We consider two subsets of ECoG data for each patient: a segment of on-going inter-ictal data, to calculate functional connectivity, and the segment with SPES data.

**Table 1:** Patient characteristics

Pat	$f_{ii}$	Grid configuration	$N_{el}$	BW	Patient state
1	2048	F($2\times 8$; $4\times 8$), IH($1\times 8$)	56	n	Awake, agile
2	512	F($4\times 8$; $4\times 8$)	56	n	Awake, quiet
3	2048	F($4\times 8$), T($4\times 8$), C($1\times 8$), IH($1\times 8$)	72	y	Light sleep
4	512	T($6\times 8$; $1\times 8$; $1\times 8$), F($2\times 8$)	58	n	Light sleep
5	2048	T($2\times 8$), C($4\times 8$)	45	y	Awake
6	512	T($6\times 8$; $2\times 8$; $1\times 8$; $1\times 8$), F($2\times 8$)	89	y	Awake, language task

$f_{ii}$: sample frequency inter-ictal ECoG in Hz, grid configuration: size and location (F: frontal, T: temporal, C: central, IH: inter-hemispheric) of the implanted electrodes, $N_{el}$: number of selected electrodes, BW: Broca’s and Wernicke’s area covered by the grid (y: yes, n: no), patient state: state of the patient during inter-ictal recording

The segments of on-going ECoG data have been recorded just preceding SPES. In this way we are sure that effects of anti-epileptic drugs and situational confounders are similar for the ongoing ECoG and SPES recordings, while any influence of SPES on the connectivity for CC and GC is excluded. We note that by imposing this condition it was not possible to control the cognitive state of the patient as this is a retrospective study. The inter-ictal ECoG segment is sampled at either $512~\text {Hz}$ or $2048~\text {Hz}$ (see Table 1). An expert clinical neurophysiologist (FSSL) marked artefacts in the raw ECoG recordings, e.g. those arising from the reference electrode. In the next sections we explain how CC and GC networks are obtained from this data. Both methods require specific pre-processing steps. For example, for CC it is usual to band-filter the data, while for GC this is not recommended (Barnett and Seth 2011). Also, it is common to apply differencing before calculating GC, while this is not the case for CC.

The protocol for SPES has been described in (van’t Klooster et al. 2011). Specifically, ten monophasic electrical stimuli are applied to pairs of horizontally adjacent electrodes. The stimuli have a duration of $1~\text {ms}$ with an inter-stimulus time of $5~\text {s}$ and an intensity of $8~\text {mA}$. During SPES ECoG data has been registered at a sampling rate of $2048~\text {Hz}$.

For all selected patients, the ECoG grid consisted of one or two large grids, spatially arranged in four or six times eight electrodes, and some additional strips consisting of eight electrodes each. We discarded all data from electrodes not used to stimulate with SPES as well as dysfunctional electrodes. Table 1 shows the selected number of electrodes per patient.

### Cross-Correlation

CC networks are non-directional weighted networks constructed from ongoing inter-ictal ECoG data. For consistency, all ongoing ECoG data are resampled to $512~\text {Hz}$ if necessary. We band-pass filtered the data to the $\theta$-, $\alpha$- and $\beta$-band, i.e. between 4 and $30~\text {Hz}$, following (Sinha et al. 2014). Next, we divided all segments of ECoG data without artefacts into non-overlapping epochs of 20 s (starting from the beginning of each segment and neglecting remaining parts or segments of < 20 s). We selected the last 60 epochs, so 20 min in total, for further analysis. For each of the selected epochs we proceed as follows for every pair of electrodes. First, we estimate the cross-correlation function for all time lags *m* with $\left| m\right| \le M$ and *M* the maximal lag in samples. Next, we set the connection strength as the maximum absolute value of this estimated cross-correlation function. We take a maximal lag of $M=26$ samples corresponding to a time of $50~\text {ms}$. We average over all 60 epochs to obtain the mean connectivity.

### Granger Causality

GC networks are constructed from the same inter-ictal ECoG data as CC networks. In contrast to CC networks, GC networks are directional. The main idea behind GC is that a connection from *x* to *y* is present if the prediction of the time series of *y* improves significantly by incorporating the past of the time series of *x* (Bressler and Seth 2011; Ding et al. 2006). In this study we use conditional GC, a multivariate form of GC, which besides the past of the time series *x* and *y* also uses the past of all other time series to determine the connectivity from *x* to *y*. This method reduces spurious connectivity, e.g. connections that arise due to common input (Barnett and Seth 2014).

GC relies on fitting multivariate autoregressive models (MVAR models) to the data. The model order *m* of this MVAR model determines the length of the history taken into account and must be specified. If we want to capture a history of $50~\text {ms}$ at a sampling rate of $512~\text {Hz}$, as in "Cross-correlation" section, we would need $m=26$. For such high model orders many unknowns must be estimated in the MVAR model. To avoid overfitting of the model, enough data points and as a consequence long time series must be considered. For such long time series the assumption of (approximate) stationarity is likely to fail. By downsampling the required model order can be reduced, while a longer history can be taken into account (Murin et al. 2016, 2018).

Our complete procedure to calculate GC is as follows. First, we resample the ECoG data to $128~\text {Hz}$. Next, first-order differencing is applied to enhance stationarity (Seth 2010). We select 60 epochs of 20 s in the same way as we do for CC (actually the same). Next, we calculate conditional GC in the time domain using the MVGC toolbox (Barnett and Seth 2014). We set the model order to $m=7$, which is sufficient to capture $50~\text {ms}$ of history. Statistical significance is assessed using the recommended options of the MVGC toolbox, i.e. Granger’s F-test with a significance level of 0.05 and the false discovery rate method to account for multi-hypothesis testing. For each epoch this results in a binary matrix with an entry being one if GC finds a significant connection and zero otherwise. Finally, we obtain the mean connectivity by averaging over all 60 epochs. The resulting network is directional with weights between zero and one.

### SPES Network

SPES networks are constructed using ERs. First, the ERs are detected from ECoG data using an automatic detector (see Supplementary Material 1). This detector determines whether an ER is present for every combination of stimulation pair and response electrode. Next, the SPES network is constructed. As for the CC and GC networks every node in this network represents an electrode. A connection from node *k* to *l* is present if at least one ER is detected at electrode *l* after any stimulation involving electrode *k*. The resulting network is directional and has binary weights.

### Localizing Broca’s and Wernicke’s Area

In three patients both the areas of Broca and Wernicke have been covered by the electrode grid. As part of clinical routine the precise locations of those two areas have been determined using electrocortical stimulation mapping (ESM). In ESM pulse trains of 4–7 s, $50~\text {Hz}$, 0.2–0.3 ms, 4–15 mA (stimulation amplitude was altered to avoid afterdischarges) are applied during a picture naming task and in case of Wernicke’s area also during item presentation in a Token Test. If repeated stimulation interferes with language (either inability to name or understand, or paraphasia) and the cause is not anarthria (sound production is unaffected) the stimulated electrode pair is marked as positive for language. Stimulations are applied to horizontally and, in contrast to SPES, also vertically and diagonally adjacent electrode pairs. An individual electrode is marked positive if it was part of at least two positively marked pairs.

### Comparing Networks

To compare CC and GC with SPES connectivity we need to cast the networks in the same form. We obtain binary CC and GC networks by thresholding the edge weights; if the weight of an edge exceeds this threshold, then there is a connection in the dichotomized network. The threshold $h^{*}$ is determined using a data-driven approach. This data-driven approach is inspired by both (Rummel et al. 2015) and the definition of outliers in a boxplot. Let $Q_{1}$ and $Q_{3}$ denote the first and third quartile of the set of all edge weights. Then $Q_{3}-Q_{1}$ denotes the inter-quartile range, which is a measure for the spread. We set $h^{*}:=\max (Q_{3}+w(Q_{3}-Q_{1}),0.1)$ with *w* a parameter. We use $w=1.5$, which is the standard choice for defining outliers (Rummel et al. 2015).

The dichotomized GC network and the SPES network are both directional, unweighted networks and hence they can be compared. A non-directional variant of the SPES network is constructed by putting an edge between nodes *i* and *j* if either $i\rightarrow j$ or $j\rightarrow i$ is present in the directional SPES network. This non-directional SPES network can be compared with the dichotomized CC network.

Next, we test if edges of the CC and GC networks coincide with those in the SPES network using a hypergeometric test for overrepresentation. Under the null hypothesis the connections of the functional network are distributed proportionally over existing and non-existing SPES connections. This hypothesis will be tested against the alternative hypothesis that CC/GC connections are overrepresented in the set of SPES connections. In other words, we test whether it is more likely to find a CC/GC connection between two nodes if there is a SPES connection between these two nodes.

The probability of finding *k* CC/GC connections in a set of $n_{s}$ SPES connections (and consequently $n_{s}-k$ non-existing CC/GC connections) is, under the null hypothesis, given by a hypergeometric distribution:

$$\begin{aligned} p_{n_s,n_{f}}(k)=\left. \genfrac(){0.0pt}0{n_{f}}{k}\genfrac(){0.0pt}0{n-n_{f}}{n_{s}-k}\Bigg /\genfrac(){0.0pt}0{n}{n_{s}}\right. , \end{aligned}$$

with $n_f$ the total number of CC/GC connections and *n* the total number of possible connections. We have $n=N_{el}(N_{el}-1)$ for the comparison between GC and SPES and $n=N_{el}(N_{el}-1)/2$ for the comparison between CC and SPES. Let $n_{sf}$ denote the number of connections in both the SPES and the CC/GC network. Under the null hypothesis, the probability *P* to have $n_{sf}$ or more CC/GC connections in the set of SPES connections is given by:

$$\begin{aligned} P=\sum _{k=n_{sf}}^{\min \left\{ n_{s},n_{f}\right\} } p_{n_{s},n_{f}}(k). \end{aligned}$$

We will reject the null hypothesis if $P<0.01$.

We also investigate the dependence of our results on the threshold for CC/GC. Let *h* be the threshold for the CC or GC network. Take $a_{c}(h)$ as the fraction of positive agreement between the SPES and CC/GC network, i.e. the number of connections that arise in both the SPES and the CC/GC network dichotomized using threshold *h* divided by the number of SPES connections. If $a_{c}$ is one all connections in the SPES network are also part of the CC/GC network. If $a_{c}$ is zero then none of the SPES connections are part of the CC/GC network. Equivalently, define $a_{nc}(h)$ as the fraction of negative agreement, i.e. the number of non-existing SPES and CC/GC connections as a fraction of the total number of non-existing SPES connections. If $a_{nc}$ is one then all non-existing SPES connections are also non-existing in the CC/GC network in which case all connections in the CC/GC network are part of the SPES network. Further, we calculate the total agreement, i.e. the number of agreeing connections and non-existing connections as fraction of the total number of possible connections. We define $h_{ma}$ as the threshold maximizing the total agreement.

Finally, we study connectivity between electrodes in Broca’s and Wernicke’s area in all three networks. We examine the number of connections found between both areas as a fraction of $n_{bw}$, the maximal number of possible connections between electrodes in Broca’s and Wernicke’s area. For the directional networks, i.e. SPES and GC, $n_{bw}$ is given by $2n_bn_w$ and for the CC network by $n_bn_w$, where $n_b$ and $n_w$ denote the number of electrodes in Broca’s and Wernicke’s area respectively.

## Results

### Cross-Correlation and SPES

**Fig. 1:**
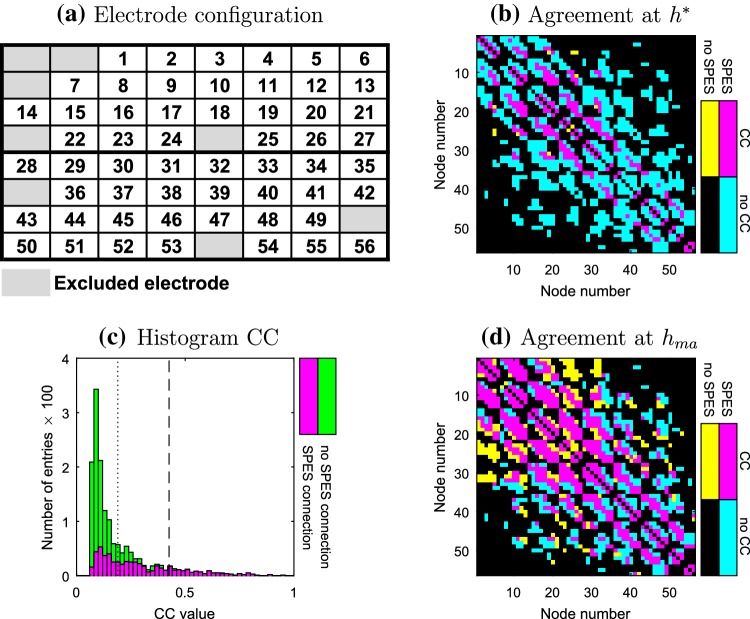
Patient 2 **a** Schematic layout of the electrode grid. **b**, **d** Comparison of the adjacency matrices of the SPES and CC network for threshold $h^{*}$ and $h_{ma}$ respectively. The numbers of the electrodes correspond to the layout in (**a**). **c** Histogram of the distribution of the CC connections. The dashed and dotted lines indicate the thresholds $h^{*}$ and $h_{ma}$ respectively

In Fig. 1b the agreement and disagreement between the adjacency matrices of the (non-directionalized) SPES and (dichotomized) CC network for patient 2 is shown (see Supplementary Material 2 for others). Observe that CC connections are located mainly at the sub-diagonals of the connectivity matrix that are directly next to the diagonal or eight columns away from the diagonal. Physically these entries correspond to the nearest neighbours of an electrode. In contrast, SPES connections are also found between more distant nodes. Further, almost all of the CC connections are contained in the SPES network, while the reverse is not true. So, the strong CC connections form a subnetwork of the non-directionalized SPES network. Adding surrogate testing to quantify the significance of CC connections as in (Rummel et al. 2011) yields the same results (results not shown).

The latter effect is robust with respect to changes in the threshold for the correlation network as indicated in the histogram in Fig. 1c displaying the distribution of CC strengths. The distribution is somewhat skewed, with a peak around 0.1 and a long tail towards the higher correlation values. The peak consists mostly of pairs of nodes that are not connected in the SPES network, while the tail is almost completely constituted by SPES connections. If we therefore slightly change the threshold for the CC network, then the dichotomized CC network would still be contained almost entirely in the SPES network.

The latter is not the case anymore if the threshold is set to $h_{ma}$, for which the agreement between the networks is maximal (see Fig. 1d). As $h_{ma}$ is smaller than $h^{*}$ more connections are included in the CC network. The additional agreement comes at the expense of adding many more non-SPES connections to the CC network.

**Fig. 2:**
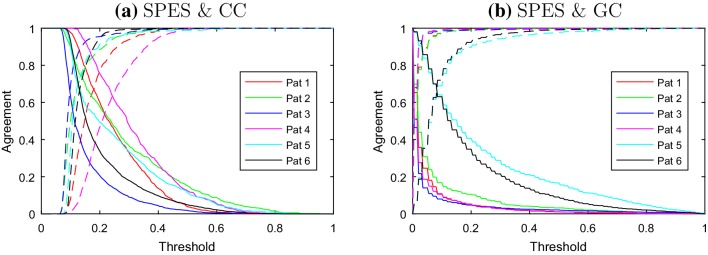
$a_c$ (solid) and $a_{nc}$ (dashed) as function of the threshold for **a** SPES and CC and **b** SPES and GC for all patients

The observations above apply to all patients. In Table 2 the results of the statistics for overrepresentation are shown. In all six patients the *P*-values are small and hence CC connections are overrepresented in the SPES network. Figure 2a depicts the dependence on the threshold for CC in relation to the agreement with the SPES network. We observe that $a_{c}$ increases if the threshold of the CC network is lowered, while on the other hand $a_{nc}$ remains close to 1 for a relatively large range of thresholds. This observation means that for a broad range of thresholds the CC network is contained almost entirely in the SPES network. On the other hand, the network induced by CC contains between 20 and 45% of the connections of the SPES network for a negative agreement, $a_{nc}=0.95$. So the CC network forms only a part of the SPES network.

**Table 2:** Summary of statistics for comparison of CC and SPES

Pat	$h^{*}$	*n*	$n_{s}$	$n_{f}$	$n_{sf}$	P
1	0.44	1540	658	56	54	$1.7\times 10^{-18}$
2	0.43	1540	566	134	128	$7.0\times 10^{-52}$
3	0.23	2556	1535	283	246	$1.3\times 10^{-25}$
4	0.55	1653	519	47	45	$3.2\times 10^{-21}$
5	0.55	990	648	59	55	$3.0\times 10^{-7}$
6	0.32	3916	2600	415	406	$7.3\times 10^{-64}$

### Granger Causality and SPES

**Fig. 3:**
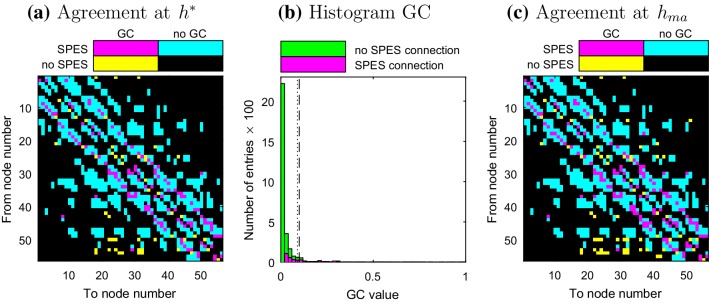
Patient 2 **a**, **c** Comparison of the adjacency matrices of the SPES and GC network for thresholds $h^{*}$ and $h_{ma}$ respectively. The numbers of the electrodes correspond to the layout in Fig. 1a. **b** Histogram of the distribution of the GC connections. The dashed and dotted lines indicate the thresholds $h^{*}$ and $h_{ma}$ respectively

Figure 3a shows the level of agreement between the adjacency matrices of the SPES and (dichotomized) GC network for patient 2 (see Supplementary Material 2 for others). Like CC connections, GC mainly finds connections between geometrically close nodes. Approximately 70% of the detected GC connections are part of the SPES network. A histogram containing the distribution of GC strengths is displayed in Fig. 3b. This distribution has its maximum at 0 and decays quickly. The thin tail of the distribution is mostly constituted by SPES connections.

The observations above apply to all patients and suggest that GC connections are overrepresented in the SPES network. It follows from the statistical test that this overrepresentation is indeed the case as can be seen in Table 3. The dependence on the threshold for GC in relation to the agreement with the SPES network can be found in Fig. 2b. Although the effect is weaker than in the CC case, $a_{c}$ increases if the threshold of the CC network is lowered, while $a_{nc}$ remains close to one for a relatively large range of thresholds. For patient 1 approximately $12\%$ of the SPES connections are part of the GC network for $a_{nc}=0.95$, in the other patients this is much higher and varies between 20 and 25%.

**Table 3:** Summary of statistics for comparison of GC and SPES

Pat	$h^{*}$	*n*	$n_{s}$	$n_{f}$	$n_{sf}$	P
1	0.10	3080	969	165	105	$2.3\times 10^{-18}$
2	0.10	3080	825	205	138	$2.5\times 10^{-36}$
3	0.10	5112	2193	219	175	$2.6\times 10^{-30}$
4	0.10	3306	791	107	83	$1.3\times 10^{-32}$
5	0.45	1980	980	209	181	$1.6\times 10^{-32}$
6	0.37	7832	3739	636	550	$9.1\times 10^{-101}$

### Broca–Wernicke Connectivity

**Fig. 4:**
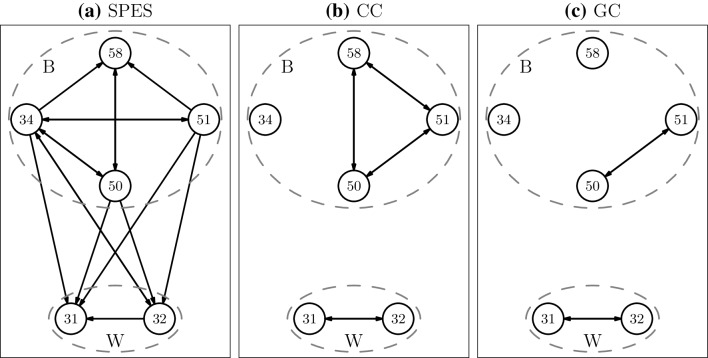
Connectivity between nodes in Broca (B) and Wernicke (W) for patient 3 inferred by **a** SPES, **b** CC and **c** GC. CC and GC networks are dichotomized using threshold $h^{*}$

**Fig. 5:**
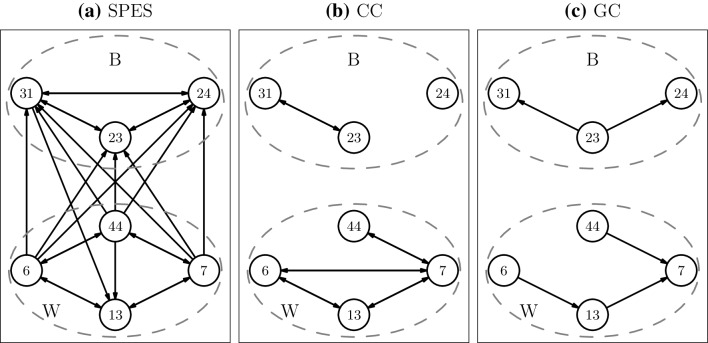
Connectivity between nodes in Broca (B) and Wernicke (W) for patient 5 inferred by **a** SPES, **b** CC and **c** GC. CC and GC networks are dichotomized using threshold $h^{*}$

**Fig. 6:**
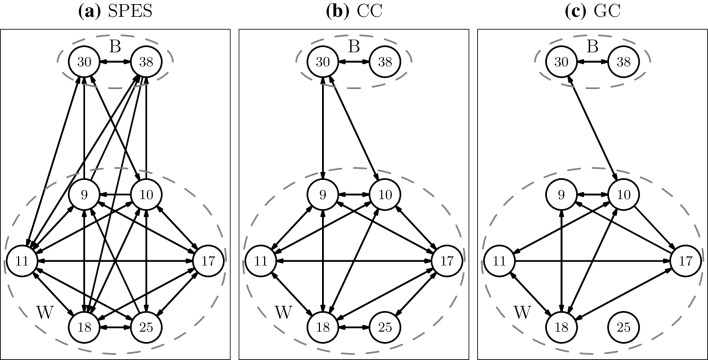
Connectivity between nodes in Broca (B) and Wernicke (W) for patient 6 inferred by **a** SPES, **b** CC and **c** GC. CC and GC networks are dichotomized using threshold $h^{*}$

The electrode grids of patients 3, 5 and 6 covered both Broca’s and Wernicke’s area. In Figs. 4, 5 and 6 the SPES, CC and GC networks restricted to the electrodes in these areas are displayed for these patients. For patient 3 Broca’s area consists of four electrodes. Three of them (50, 51 and 58) are neighbouring electrodes, while 34 is located further away, i.e. 2 cm away from 50 (see Supplementary Material 2 for the schematic location of the electrodes). Wernicke’s area is covered by two neighbouring electrodes. In Broca’s area 67% of all possible connections are found in the SPES network, while for CC and GC this is 50% and 17% respectively. In both the CC and GC network electrode 34 is isolated, which agrees with our previous finding that those methods find predominantly close-by connections. SPES, however, finds connections to this more distant node. On the other hand, electrodes 50 and 51 are reciprocally connected in both the CC and GC network, while there is no edge in the SPES network between those two electrodes. The absence of SPES connections is partly a consequence of how we build the SPES network. As electrode 51 is located at the end of a row in the grid it is part of only one stimulation pair, namely 50–51, therefore it is impossible to find a connection from 51 to 50 in the SPES network. The reverse connection was also not found as electrode 51 became saturated when stimulating electrode pair 49–50. The two electrodes in Wernicke’s area are reciprocally connected in the CC and GC network, while SPES could not recover the connection from 31 to 32 as the only stimulation pair containing electrode 31 is the pair 31–32. Connectivity between Broca’s and Wernicke’s areas is only found in the SPES network. All the connections except one are from Broca’s to Wernicke’s area.

For patient 5, Broca’s and Wernicke’s areas consist of 3 and 4 neighbouring electrodes respectively (see Supplementary Material 2 for the location of the electrodes). The electrodes in Broca’s area are fully connected to each other in the SPES network. In the CC and GC networks we find only a third of all possible connections in Broca. In Wernicke’s area the SPES network is also well-connected, containing 75% of all possible connections. The CC network has a relatively high internal connectivity, as 2/3 of all possible connections are present. In the GC network on the other hand, only a quarter of all possible connections are found. For patient 5, as for patient 3, only the SPES network shows connectivity between Broca’s and Wernicke’s area, however the orientation is reversed; all connections except one are directed from Wernicke’s to Broca’s area.

For the last patient, 2 electrodes were placed on Broca’s area while there were 6 on Wernicke’s area. In all three networks the 2 electrodes in Broca’s area were reciprocally connected. In Wernicke’s area the SPES network contains 93% of all possible connections. The CC and GC are also relatively well-connected finding 80% and a half of all possible connections respectively. In contrast to the other two patients some CC and GC connections are found between Broca’s and Wernicke’s area. For GC this is the reciprocal connection between electrodes 10 and 30, which is 8% of all possible connections. The CC network also contains a connection between 9 and 30, yielding a total of 17% of all possible connections. These percentages are still much lower than the SPES network which finds one third of all possible connections from Broca’s area to Wernicke’s area and one half in opposite direction.

## Discussion

### Network Comparison

We have compared connectivity derived from SPES to CC and GC networks derived from on-going inter-ictal ECoG. All three methods yield primarily nearest neighbour connections, however SPES networks are usually connected more densely and include more distant connections than CC and GC networks. We find a strong relationship between CC and SPES networks, i.e. strong CC connections form a subset of the SPES network. The relation between SPES and GC networks is weaker. Although GC connections coincide more frequently with SPES connections compared to non-existing SPES connections, they do not form a subset of the SPES network.

One of the most important factors underlying the difference between the SPES and CC/GC networks is the process by which networks are inferred. Non-evoked connectivity methods try to recover connectivity from passive observations using statistical dependencies between the time series of nodes. Long range connections may have small ongoing influences that are easily overpowered by activity of local circuits and their common input. CC and GC will therefore find only weak coupling between such nodes, as has been observed in a computational modeling study (Ponten et al. 2010). This phenomenon might be typical for the centimeter-scale at which ECoG is recorded. In SPES, connections are activated strongly, due to the electrical stimulus. This causes a large response at the receiving electrode, which makes it distinguishable from the ongoing activity. This might explain the difficulty of detecting long-range connections among networks based on ongoing ECoG activity, compared to SPES networks.

In our study, all three constructed networks use the electrodes of the ECoG grid as nodes which enables a straightforward comparison between the networks. Moreover, as all three networks represent local connectivity their scale is equal. Other studies have compared SPES to larger scale networks inferred using whole-brain imaging modalities like diffusion weighted imaging (DWI) and resting-state functional magnetic resonance imaging (fMRI). A higher overlap than expected by chance was found between edges of dichotomized DWI and SPES networks (Parker et al. 2018). On the other hand, the correlation between connection strengths taken over the whole brain is low both for comparing SPES with DWI (Jones et al. 2014; Donos et al. 2016b; Parker et al. 2018) and fMRI (Keller et al. 2011; Jones et al. 2014). There are however clusters of brain regions that have similar connectivity in networks constructed with SPES as well as DWI (Donos et al. 2016b) and fMRI (Keller et al. 2011).

An example of such a cluster is the language circuit, in which a strong relation was found between DWI and SPES amplitude and latency (Conner et al. 2011). This relation is in accordance with earlier studies on DWI (Catani et al. 2005) and CCEP (Matsumoto et al. 2004) in the language circuit. Similar results were found for a comparison between the amplitude of CCEP and resting-state fMRI (Keller et al. 2011). Here it was found that CCEP connections starting from Broca, Wernicke or sensory-motor regions show a much higher resting-state fMRI connectivity compared to non-existing CCEP connections starting from these regions.

The classical language circuit was the first functional network studied with CCEP (Matsumoto et al. 2004). Bidirectional connectivity between Broca’s and Wernicke’s area was found, in contrast to the traditional Wernicke–Geschwind model, which hypothesized only connectivity from Wernicke’s to Broca’s area (Dronkers et al. 2000). Stimulation of electrodes in Wernicke’s area elicits less well-pronounced responses in electrodes in Broca’s area compared to the other way around. These results have been confirmed by multiple CCEP studies (see Matsumoto et al. 2017 for an overview). Our findings are in accordance with these results. However, in patient 3 the connections are predominantly from Broca’s to Wernicke’s area, while in patient 5 the reverse holds. This result could be a consequence of our definition of Broca’s and Wernicke’s area, namely as electrodes that are part of at least two stimulation pairs marked positive for language using ESM.

In contrast to the SPES network, we find that CC and GC networks reveal only some connectivity between Broca’s and Wernicke’s area in one of the three patients. This result could be expected because those two methods yield mostly local connections. Another explanation could be the state of the patient during ECoG acquisition. The only patient for which CC and GC reveal connections between Broca and Wernicke, patient 6, was busy with a language task during the ongoing ECoG recordings. One could therefore expect that Broca’s and Wernicke’s area were more active. Nevertheless, CC and GC find only a fraction of the connections that are found with SPES.

### Methodological Issues

There are many methodological issues in constructing functional networks from on-going ECoG starting already with pre-processing of the ECoG data. First of all, there are multiple options for referencing ECoG data. We used a common reference montage with an extracranial reference electrode located on the contralateral mastoid. A common reference montage has the disadvantage that noise on the reference electrode affects all channels of the recorded ECoG data. For an extracranial reference this is more problematic than for an intracranial reference, as the former is more susceptible to pick up muscle artefacts. Unfortunately, it was not possible to use an intracranial reference electrode as we do not apply epidural reference electrodes during grid recordings. To reduce the influence of common reference noise we removed the parts of the recordings in which common reference artefacts were visible according to an expert clinical neurophysiologist (FSSL). Further, the common reference noise will increase the level of background correlations which may lead to spurious connections (Bastos and Schoffelen 2015). We therefore used the data-driven threshold $h^{*}$ which ensures that only correlations that are sufficiently above the background level will be included, at the cost of a less extended dichotomized network. As an alternative to a common reference montage bipolar and average reference montages may be used. These montages try to remove the common reference noise by taking linear combinations of the signals, but this changes the interpretation of the nodes and, moreover, it creates linear dependencies between the signals. The latter proved to be problematic when we tried to calculate multivariate GC. Given these practical constraints, this is the only approach allowing us to compare the networks.

Another methodological issue is the length of the epochs for calculating functional connectivity. If those epochs are too long the time series might not be stationary. On the other hand, the epochs should be long enough to reliably infer the connectivity. In the case of CC for example, two finite independent time series may show a high correlation, although this is theoretically zero for infinite ones (Rummel et al. 2015). In this study we took epochs of 20 s as preliminary investigations showed the CC values to stabilize for longer epochs. For consistency we used the same epoch length for GC.

We observed that CC is a robust functional connectivity measure. GC in contrast, is much more sensitive to non-stationarity of the time series and small artefacts. This sensitivity might be because GC is a noise-driven method which needs a certain amount of stochasticity in the data. In ECoG recordings this stochasticity can be too small causing the factors mentioned above to dominate the time series (Barnett and Seth 2014).

The SPES networks we constructed are based on ERs found with our automatic detector. The principle of the detector is straightforward and uses the amplitude of responses relative to the baseline to qualify a response as ER or not. Similar principles are applied in other studies (Lacruz et al. 2007; David et al. 2013; Entz et al. 2014). We have validated our detector on visually classified responses (see Supplementary Material 1). An alternative for our binary classification is to use the amplitude of the response itself to infer the strength of a connection as is often used in CCEP studies (Matsumoto et al. 2004; Conner et al. 2011; Enatsu et al. 2012). The amplitude, however, depends on multiple factors including how well an electrode makes contact to the cortex. Alternatively, a variable amplitude protocol may be used to infer connection strengths for SPES (Donos et al. 2016a, b).

Another potential problem in the construction of SPES networks is the effect of volume conduction (VC) (Shimada et al. 2017). Due to the direct and artificial nature of the stimulation, a large source of neuronal activity can be generated which might be picked up by electrodes surrounding the stimulation pair. Note that this phenomenon is not about VC of the electrical stimulation itself. The result could be that spurious local connectivity is found (Shimada et al. 2017). We investigated the influence of VC in Supplementary Material 3 and concluded that its effects in our SPES data are small. It is, therefore, not necessary to account for VC effects in our SPES networks.

A point of attention is the non-stationarity of brain connectivity. We chose to select ongoing ECoG data recorded just preceding SPES as the patient state during that time would be most similar to the one during stimulation. Although functional connectivity was calculated over several minutes, we noted that differences in the results were small in general. The network converged rapidly to an average structure as has been described before (Kramer et al. 2011). In contrast to functional connectivity, the presence of a SPES connection is measured over approximately one minute. One might therefore think that variation in SPES connectivity is high, however this is not the case; SPES networks are highly reproducible. In preliminary research we found an agreement in connectivity between two SPES sessions of around 75%, which is in-line with another CCEP study (Entz et al. 2014).

### Overview

The data in this study is obtained from intracranial grid recordings in patients with refractory epilepsy. In this field the study of brain networks may help to improve localization of the epiletogenic focus (Haneef and Chiang 2014; Khambhati et al. 2016). An interesting recent development is to combine networks with computational models (Terry et al. 2012; Benjamin et al. 2012; Hebbink et al. 2017). In these models, the activity of the neuronal population underlying each node is modeled by a neural mass. The nodes influence each others’ dynamics according to the connectivity of the network. By using patient-specific networks the effect of epilepsy surgery can be predicted (Goodfellow et al. 2016; Sinha et al. 2017; Jirsa et al. 2017; Lopes et al. 2017), however many challenges still remain before these methods could be applied in clinical practice (Eissa and Schevon 2017; Youngerman et al. 2017).

One of the questions in this approach is which type of network should be used. To date, computational studies have used networks derived from ongoing intracranial EEG data, both inter-ictal (Sinha et al. 2017) as well as seizure data (Goodfellow et al. 2016; Lopes et al. 2017). However, as we showed in this study, inter-ictal CC and GC networks do not capture all known anatomical connections, for instance those between Broca and Wernicke. SPES networks might be a good alternative as such models would incorporate more physiological connections. Moreover, SPES ER networks contain information about both the seizure onset zone (van Blooijs et al. 2018; Boido et al. 2014) and seizure propagation (Enatsu et al. 2012; Mouthaan et al. 2016). It would therefore be interesting to compare SPES and ictal networks.

Another issue is the limited covering of electrode grids which results in localized sampling only. In a computational network approach this limited covering leads to boundary effects. A possible solution is to incorporate a patient specific-network into a larger, generic, full-brain network. For SPES, there are some brain atlases available (David et al. 2013; Donos et al. 2016b) which could be beneficial for such studies. Seen in this light our work gives a better foundation for the use of networks in such future computational studies.

## Electronic supplementary material

Below is the link to the electronic supplementary material.

Supplementary Material 1 (PDF 2.755 MB)

Supplementary Material 2 (PDF 887 KB)

Supplementary Material 3 (PDF 460 KB)
