# Supplementary material for: A Comparison of Evoked and Non-evoked Functional Networks
Source: Brain Topogr. 2018 Dec 6;32(3):405–17. doi: 10.1007/s10548-018-0692-1 (PMC6476864; doi:10.1007/s10548-018-0692-1)
Supplement: Supplementary file 1 — Supplementary Material 1 (PDF 2.755 MB) [file 10548_2018_692_MOESM1_ESM.pdf]

A comparison of evoked and non-evoked functional networks

Supplementary material 1: Automatic ER detector

Jurgen Hebbink[1,2], Dorien van Blooijs[1], Geertjan Huiskamp[1], Frans S.S. Leijten[1], Stephan A. van Gils[2] and Hil G.E. Meijer[2]

[1]Department of Neurology and Neurosurgery, Brain Center Rudolf Magnus, University Medical Centre Utrecht, Heidelberglaan 100, 3584 CX Utrecht, The Netherlands
[2]Department of Applied Mathematics, MIRA Institute for Biomedical Engineering and Technical Medicine, University of Twente, Drienerlolaan 5, 7500 AE Enschede, The Netherlands

November 23, 2018

1 Automatic detection of ERs

In this appendix we provide a detailed description of our automatic detector (AD) for ERs. First, the detector algorithm is explained. Next, we describe our gold standard for ER detection, i.e. visual classification. Finally, we use a learning set to tune the parameters of the AD and test the AD's performance on a validation set.

1.1 Automatic detector algorithm

The basic principle of the AD is that an ER yields a local extremum within 100 ms after the stimulation that is "sufficiently" high above baseline. The input for the AD is ECoG data recorded during SPES. In our SPES protocol each stimulation pair is probed ten times with an inter-stimulus time of 5 seconds. Around each stimulus an epoch of 5 seconds starting 2 s before the stimulus is selected. We average all ten epochs and subtract the median taken over the whole interval of the averaged response.

Next, we detect the extrema in a time range of 9 ms to 100 ms after the stimulation using the Matlab function peakfinder. This function has a parameter s, specifying how much an extreme value should deviate from the neighbouring time points to qualify as an extremum. Let σ_{bl} be the standard deviation of the 2 seconds prior to stimulation. We set $\sigma = \max\{\sigma_{bl}, \sigma_{min}\}$, where σ_{min} is a user-defined parameter. If the amplitude of the extreme value is higher than a threshold $\theta\sigma$, then the AD classifies the response as an ER. This procedure is illustrated in Figure 1(b) and (c).

1.2 Performance of the automatic detector

We use visual classification of ERs as gold standard for ER detection. ERs were annotated in Micromed, SystemPlus Evolution by one observer (DvB). ECoG data was visualized

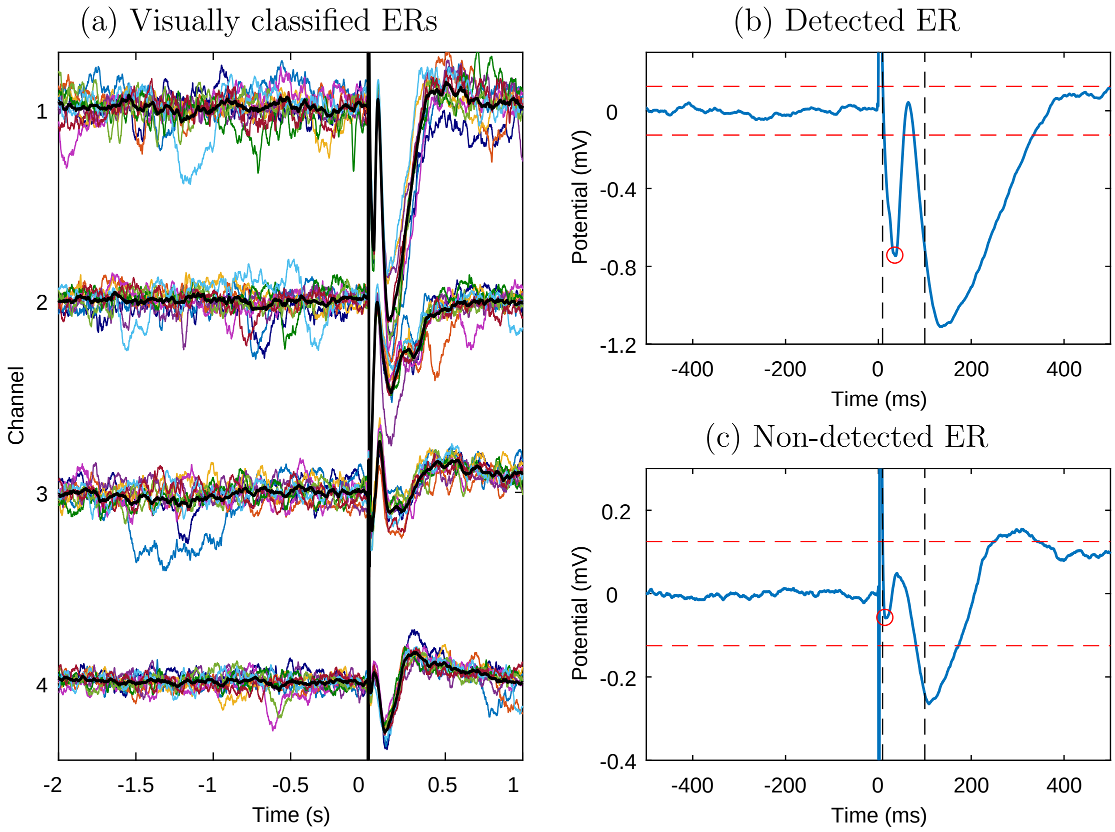

Figure 1: (a) Examples of visually classified ERs. The thin coloured lines indicate the responses to single stimulation trials, while the thick black lines indicate the averaged responses over all ten trials. The responses for channels 1, 2 and 3 were also classified as an ER by the automatic detector. (b) and (c) The blue line shows the magnification of the average response around the stimulation of channels 1 and 4 respectively. The dashed red lines indicate $\pm\theta\sigma$, the threshold for detecting an ER. A response is classified as ER if the amplitude of the detected peak exceeds the threshold in the time interval between the dashed black lines which is the case for (b) but not for (c).

with 5 s/page and a variable scaling (usually 1200 μV/cm), depending on the amplitude of the signals and the number of implanted electrodes. If amplitudes were too high to be able to differentiate ERs in neighboring electrodes, the scaling was decreased to 2000 μV/cm. No additional software filtering was used. Figure 1(a) shows some examples of visually classified ERs. In total we have annotated data of six patients, which we equally divide into a learning and validation set. The SPES data of two patients is also used in the main part of this work, i.e. patients A3 and A4 correspond to patients 3 and 4, respectively.

The data of the learning set is used to tune the three parameters of the AD: s, σ_{min} and θ. We vary s between 10 and 200 μV with steps of 10 μV, σ_{min} between 0 and 100 μV with steps of 10 μV and θ between 1 and 15 with step size 0.5. For each parameter combination we determine the true positives (visually annotated ERs detected by the AD) and the true negatives (responses not classified as ER by both visual annotation and AD). From this we calculate the true positive rate, tpr, as the number of true positives divided by the total number of visually annotated ERs and the true negative rate, tnr, as the number of true negatives divided by the total number of responses not classified

as ER by visual detection. In case of an ideal detector both *tpr* and *tnr* will be one. For each parameter combination we measure the quality of the AD by the distance d of *tpr* and *tnr* to the perfect detection:

$$d(tpr, tnr) = \sqrt{(1 - tpr)^2 + (1 - tnr)^2}.$$

We minimize d to find the optimal parameter values for the AD. The optimal parameters are a threshold θ of 2.5, $s = 20\ \mu$V and a minimal standard deviation σ_{min} of 50 μV. For these optimal parameters, we have $d = 0.23$. The performance on the learning set can be found in Table 1. This table shows, besides *tpr* and *tnr*, also the positive predicted value (*ppv*, number of true positives divided by total number of automatic detected ERs), negative predicted value (*npv*, number of true negative divided by total number of responses not classified as ER by automatic detection) and accuracy (*acc*, fraction of correctly identified responses by the AD).

We evaluate the automatic detector with the optimal parameter settings on the validation set. Also these results can be found in Table 1. The mean sensitivity and specificity are both high and vary within an acceptable range. So for each patient most of the responses classified as ER by the AD are also annotated as ERs by the visual detection and only a few of the visually annotated ERs are missed by the detector. We conclude that the performance is sufficient to construct a physiological network that is reliable for further analysis.

Set	Patient	*tpr*	*tnr*	*ppv*	*npv*	*acc*
Learning	A1	0.85	0.83	0.57	0.96	0.83
	A2	0.73	0.88	0.60	0.93	0.85
	A3	0.83	0.79	0.44	0.96	0.79
	mean	0.80	0.83	0.54	0.95	0.83
Validation	A4	0.71	0.94	0.84	0.87	0.86
	A5	0.77	0.94	0.68	0.96	0.92
	A6	0.86	0.85	0.72	0.93	0.85
	mean	0.78	0.91	0.75	0.92	0.88

Table 1: Performance of the automatic ER detector.
