# Supplementary material for: A Comparison of Evoked and Non-evoked Functional Networks
Source: Brain Topogr. 2018 Dec 6;32(3):405–17. doi: 10.1007/s10548-018-0692-1 (PMC6476864; doi:10.1007/s10548-018-0692-1)
Supplement: Supplementary file 2 — Supplementary Material 2 (PDF 887 KB) [file 10548_2018_692_MOESM2_ESM.pdf]

A comparison of evoked and non-evoked functional networks

Supplementary material 2: Results in other patients

Jurgen Hebbink[1,2], Dorien van Blooijs[1], Geertjan Huiskamp[1], Frans S.S. Leijten[1], Stephan A. van Gils[2] and Hil G.E. Meijer[2]

[1]Department of Neurology and Neurosurgery, Brain Center Rudolf Magnus, University Medical Centre Utrecht, Heidelberglaan 100, 3584 CX Utrecht, The Netherlands
[2]Department of Applied Mathematics, MIRA Institute for Biomedical Engineering and Technical Medicine, University of Twente, Drienerlolaan 5, 7500 AE Enschede, The Netherlands

October 4, 2018

1 Detailed results of other patients

In this appendix we show detailed results of comparing SPES to cross-correlation (CC) and Granger Causality (GC) for patients 1 and 3-6. Figures 1-5 show for each patient the schematic layout of the electrode configuration as well as the comparison between SPES and CC and SPES and GC in the same way as Figures 1 and 3 of the main text. The results in these patients are similar to those of patient 2, which are presented in the main part of our work.

Patient 1

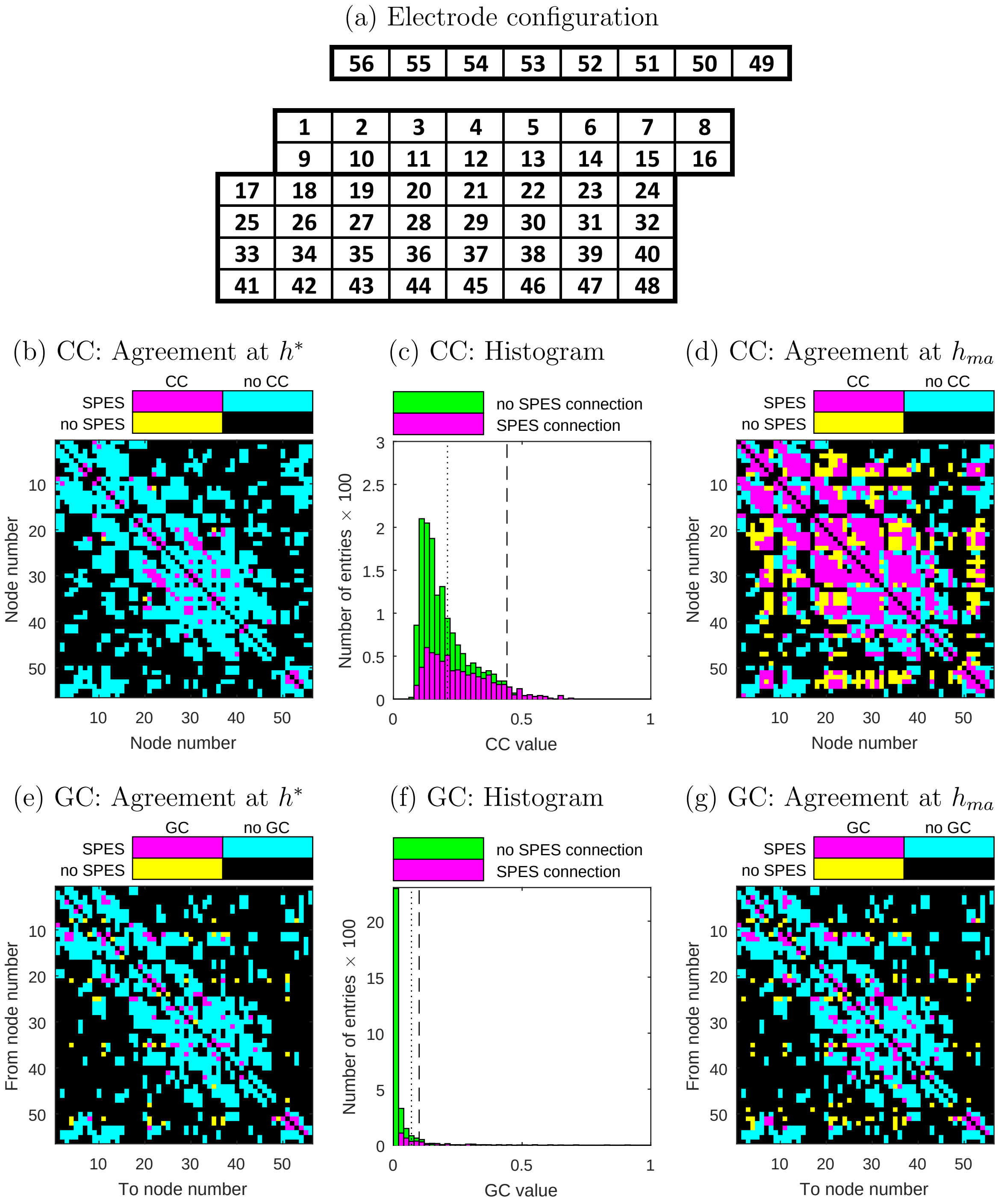

Figure 1: Results for patient 1.

Patient 3

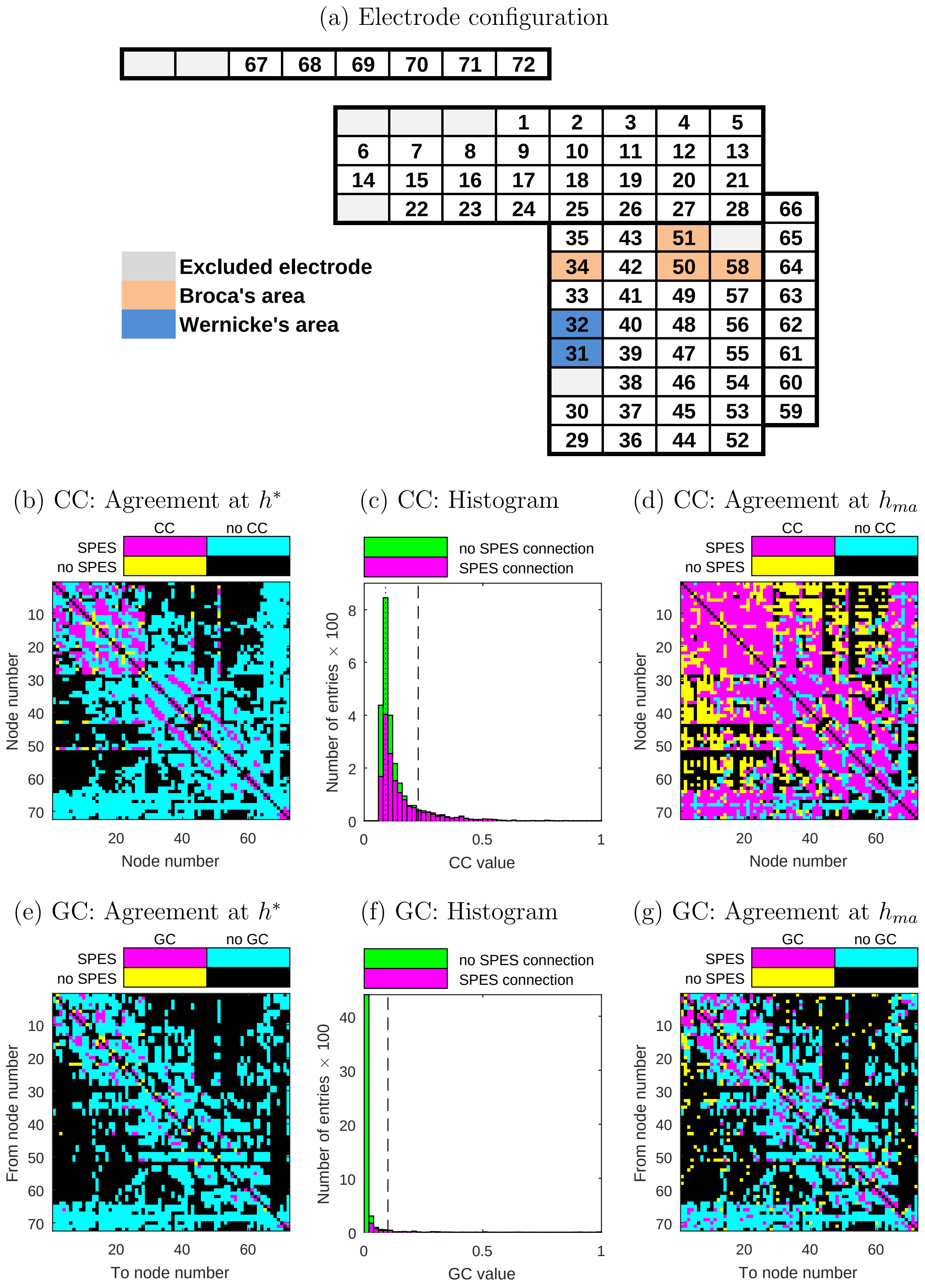

Figure 2: Results for patient 3.

Patient 4

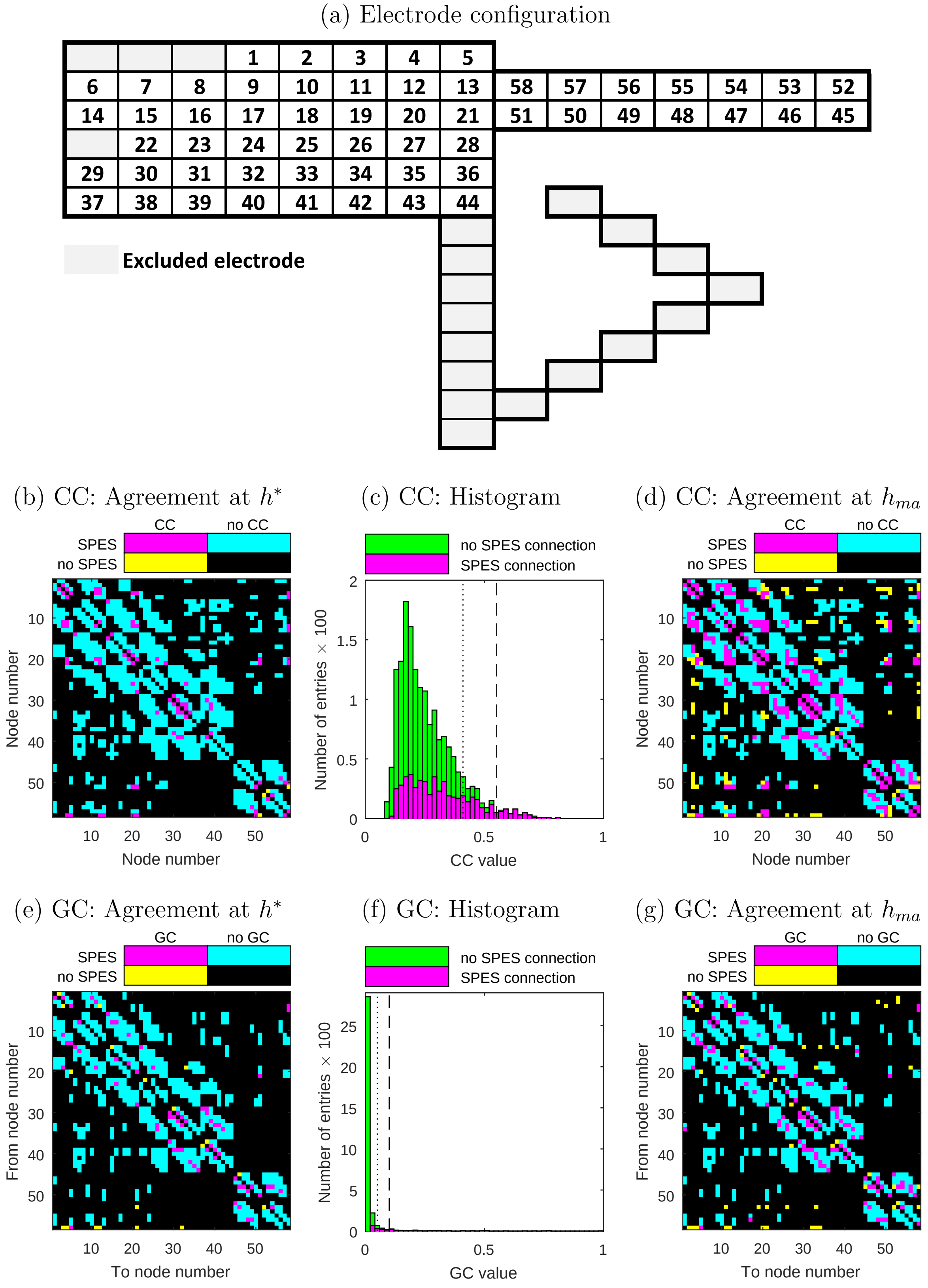

Figure 3: Results for patient 4.

Patient 5

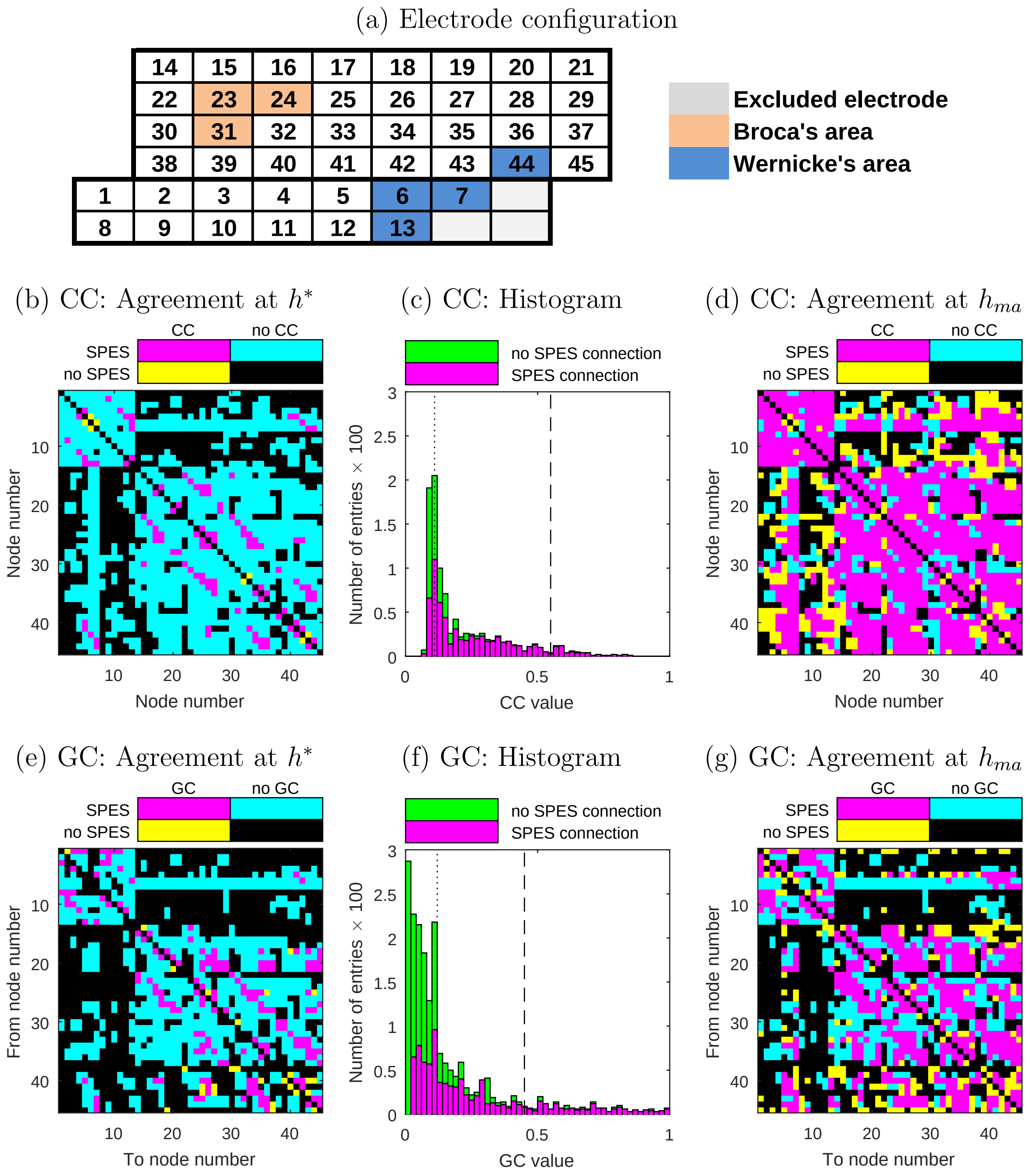

Figure 4: Results for patient 5.

Patient 6

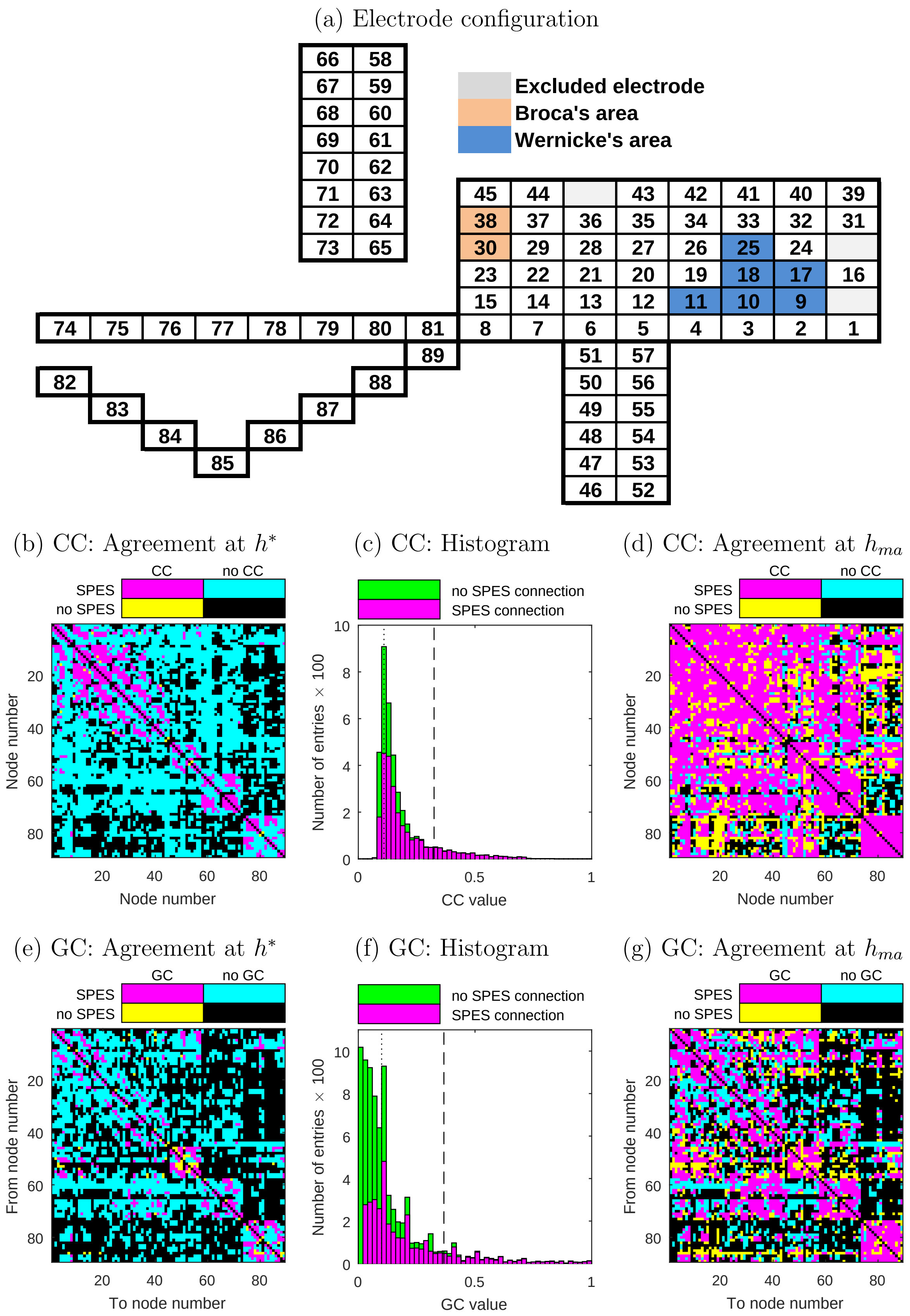

Figure 5: Results for patient 6.
