# Supplementary material for: A Comparison of Evoked and Non-evoked Functional Networks
Source: Brain Topogr. 2018 Dec 6;32(3):405–17. doi: 10.1007/s10548-018-0692-1 (PMC6476864; doi:10.1007/s10548-018-0692-1)
Supplement: Supplementary file 3 — Supplementary Material 3 (PDF 460 KB) [file 10548_2018_692_MOESM3_ESM.pdf]

A comparison of evoked and non-evoked functional networks

Supplementary material 3: SPES & volume conduction

Jurgen Hebbink[1,2], Dorien van Blooijs[1], Geertjan Huiskamp[1], Frans S.S. Leijten[1], Stephan A. van Gils[2] and Hil G.E. Meijer[2]

[1]Department of Neurology and Neurosurgery, Brain Center Rudolf Magnus, University Medical Centre Utrecht, Heidelberglaan 100, 3584 CX Utrecht, The Netherlands
[2]Department of Applied Mathematics, MIRA Institute for Biomedical Engineering and Technical Medicine, University of Twente, Drienerlolaan 5, 7500 AE Enschede, The Netherlands

October 4, 2018

1 Introduction

Volume conduction (VC) may play a role in the observation of evoked responses by single pulse electrical stimulation (SPES). Due to its direct and artificial nature, SPES can evoke a large source of neuronal activity which might be picked up by multiple nearby electrodes. In this appendix we investigate this effect by looking at the timing of the N1 peak of an early response (ER, see figure 1). The timing of the N1 across electrodes will be exactly the same if ERs arise due to VC, as this acts instantaneously.

2 Detection of N1 peaks

We take, for the same patient population as used in the main text, the ECoG data of all ERs found by our automatic detector (see supplementary material 1). For each ER we try

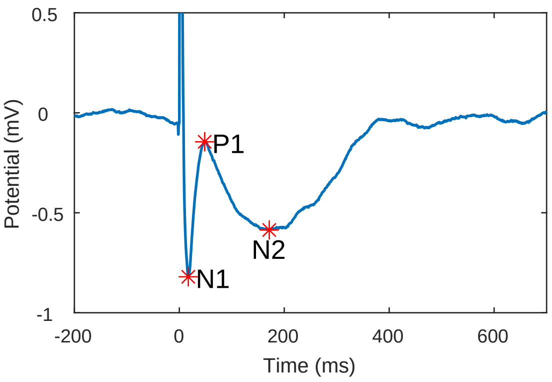

Figure 1: Example of a characteristic ER. The three peaks of the ER are called N1, P1 and N2.

to find its three characteristic peaks, i.e. the N1, P1 and N2 (see figure 1), in the ECoG data averaged over all ten stimulation trials as follows. First all positive and negative peaks in the time range 9-300 ms after the stimulation are detected using the Matlab function peakfinder. This function has a parameter s, specifying how much an extreme value should deviate from the neighbouring time points to qualify as an extremum. We put $s = 50\ \mu$V for the positive peaks and, in order to detect the less sharp N2 peaks, $s = 20\ \mu$V for the negative ones. In case there are no positive or less than 2 negative peaks the signal is discarded.

Now, let $\widetilde{P}_1 < \widetilde{P}_2 < \ldots$ be the time of the positive peaks and similarly $\widetilde{N}_1 < \widetilde{N}_2 < \ldots$ the time of the negative peaks. If $\widetilde{N}_1 < \widetilde{P}_1 < \widetilde{N}_2$ the detected peaks are in the expected order and we take these peaks as our N1, P1 and N2. Else either $\widetilde{P}_1 < \widetilde{N}_1$ or $\widetilde{N}_2 < \widetilde{P}_1$ holds. In these cases, a spurious peak is detected, e.g. due to noise or spontaneous activity. We try to correct these cases in the following way. If $\widetilde{P}_1 < \widetilde{N}_1$, we check the second positive peak, $\widetilde{P}_2$. If this peak lies between the two negative peaks, we accept $\widetilde{N}_1$, $\widetilde{P}_2$ and $\widetilde{N}_2$ as our N1, P1 and N2. Else we discard the signal. In case $\widetilde{N}_2 < \widetilde{P}_1$, we check if $\widetilde{N}_3$ appears after $\widetilde{P}_1$. If true, then $\widetilde{N}_2$, $\widetilde{P}_1$ and $\widetilde{N}_3$ are taken as N1, P1 and N2, else the signal is rejected.

3 Spread of N1 times

Figure 2 shows the N1 times for a selected stimulation. The N1 times differ much from each other and range between 13 and 34 ms with a standard deviation of 5.45 ms. Even direct neighbours of the stimulation pair show distributed N1 times. The node left of the stimulation pair (orange border) has a relatively late N1. On the other hand, the node below the right stimulation electrode (green border) has a rather early N1 and a less pronounced N2. From the difference in timing of the N1 peak one can conclude that VC does not play an important role for this stimulation pair.

A different example is shown in figure 3. Here volume conduction seems to play an important role as the spread in N1 times is small. The standard deviation in this case is only 2.45 ms. Most responses are seen approximately 18 ms after stimulation. Also the shape of the responses is very similar, especially the N1 and P1 component.

Next we investigate the effect of VC on a stimulation level. We calculate for each stimulation evoking more than three ERs, the standard deviation of the N1 times. For patient 2 the distribution of these standard deviations is shown in Figure 4a. For most stimulations the standard deviation is around 5 ms, corresponding to the situation in Figure 2. In only three cases the standard deviation is low (< 2.5 ms), one of them being the stimulation shown in Figure 3. Similar results are found in other patients as shown in Figure 5. So most ERs represent local activity, and not distant responses picked up by VC. The latter happens only in a few stimulations.

Finally, we look at the relation between the N1 times and the distance of the response electrode to the stimulation pair. Here distance is defined as the mid point of the stimulation pair. Results for patient 2 are shown in Figure 4b. Observe the rather big spread in N1 times per distance. Further, the N1 time increases slightly with distance. The correlation between N1 time and distance is 0.16 ($p = 0.007$), for all response electrodes within 3 cm of the stimulation pair. Results in other patients are comparable, see Figure 5. The correlations are 0.36 ($p < 0.001$), 0.28 ($p < 0.001$), 0.30 ($p < 0.001$), 0.15 ($p = 0.011$) and 0.33 ($p < 0.001$) for patient 1, 3, 4, 5 and 6 respectively.

If the observed ERs in SPES were mainly due to VC, then one would expect that distance would not play a role at all for the N1 times, as VC act instantaneously, and

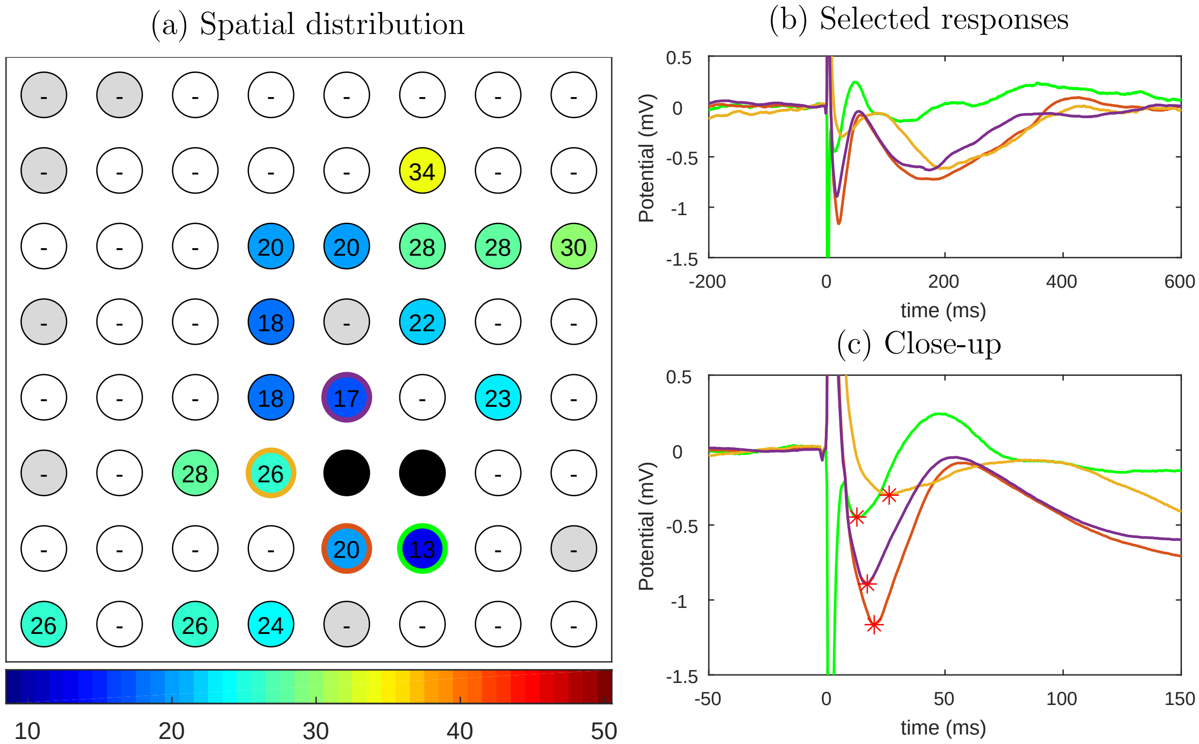

Figure 2: N1 times for a selected stimulation. (a) Spatial distribution. Numbers and colours indicate N1 time of an electrode (in ms). Stimulated and excluded electrodes are coloured black and gray, respectively. (b) Time course of the responses of four selected electrodes. Colours match with the borders of the nodes in (a). (c) Close-up of (b).

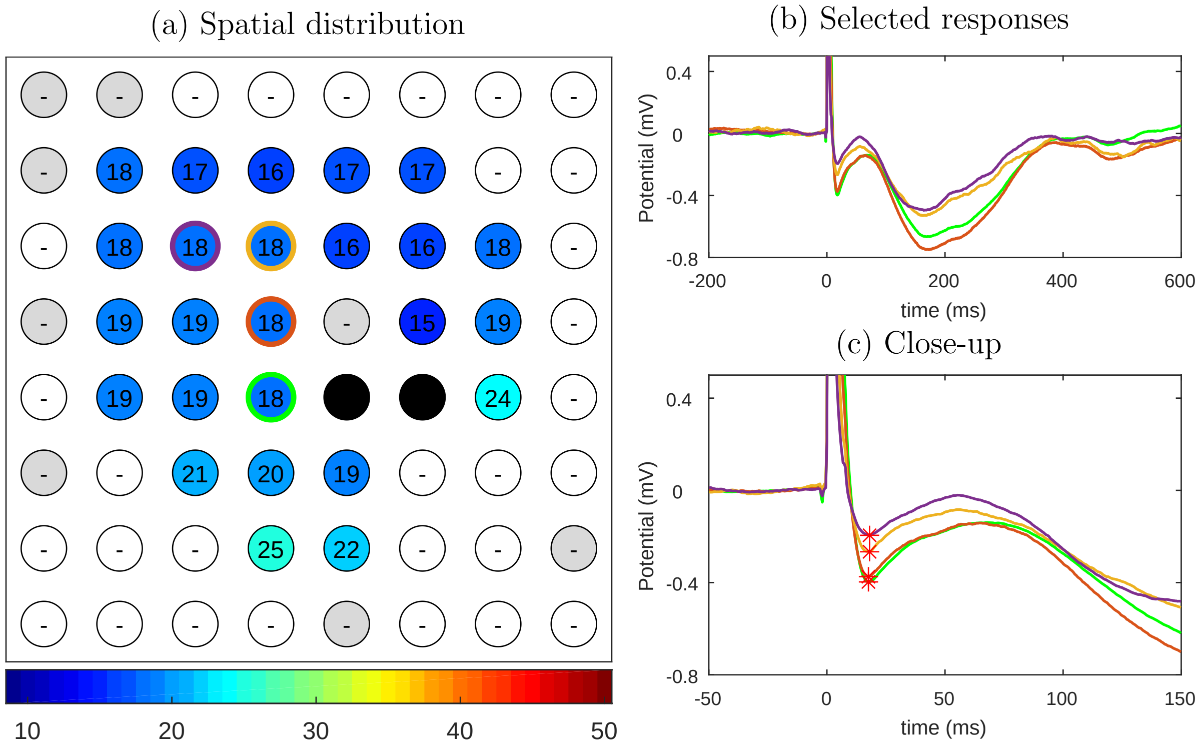

Figure 3: N1 times for a selected stimulation. (a) Spatial distribution. Numbers and colours indicate N1 time of an electrode (in ms). Stimulated and excluded electrodes are coloured black and gray, respectively. (b) Time course of the responses of four selected electrodes. Colours match with the borders of the nodes in (a). (c) Close-up of (b).

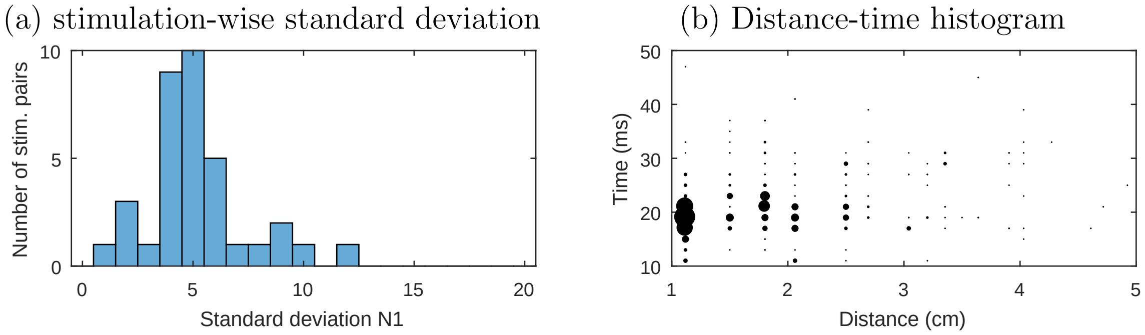

Figure 4: Distribution of the N1 peaks in patient 2. (a) Histogram of the stimulation-wise standard deviation of N1 times. (b) Histogram showing the distribution of all N1 over both distance to the stimulation pair and peak time. The size of the dots indicate the number of observations.

hence the correlation would be (almost) zero. On the other hand, if the SPES evoked activity would propagate in all directions homogeneously, then the correlation would have been much closer to one. So the distance to the stimulation pair plays a role in the observed N1 time, but is not the main determining factor.

4 Conclusion & discussion

We conclude that VC plays only a minor role in our SPES data. In only a few stimulations we noticed a (possible) VC effect as N1 peaks appeared at almost the same moment in a large number of electrodes. For most stimulations however, the N1 times were distributed, which implies that VC plays a minimal role in our recordings. The positive correlation between N1 times and the distance to the stimulation electrodes strengthens this conclusion.

Our results contradict with an earlier study, were VC in SPES was investigated using the root mean square (RMS) of the response [1]. They found that the RMS was proportional to the squared inverse of the distance to the stimulation pair and attributed this finding to VC. An alternative explanation for the observed decay in amplitude could be a distance-dependent decay of connectivity strength. We think therefore that investigating the timing of the N1 responses, like we did, is a more direct approach to investigate the VC effect.

Another explanation for the difference between our results and those of [1] can be the settings of the stimulation. We used monophasic stimulation of 8 mA and 0.1 ms. In [1] biphasic stimulation with a time of 0.15 ms per phase was used, which is much longer. Also, the biphasic stimulation might trigger different cortical activity.

We conclude that the effects of VC in our data are limited. VC therefore won't influence (nearby) connectivity in our SPES network much and it is not necessary to account for the effects of VC.

References

[1] S. Shimada, N. Kunii, K. Kawai, T. Matsuo, Y. Ishishita, K. Ibayashi, and N. Saito. "Impact of volume-conducted potential in interpretation of cortico-cortical evoked potential: Detailed analysis of high-resolution electrocorticography using two

mathematical approaches". *Clinical Neurophysiology* 128.4 (2017), pp. 549–557. DOI: 10.1016/j.clinph.2017.01.012.

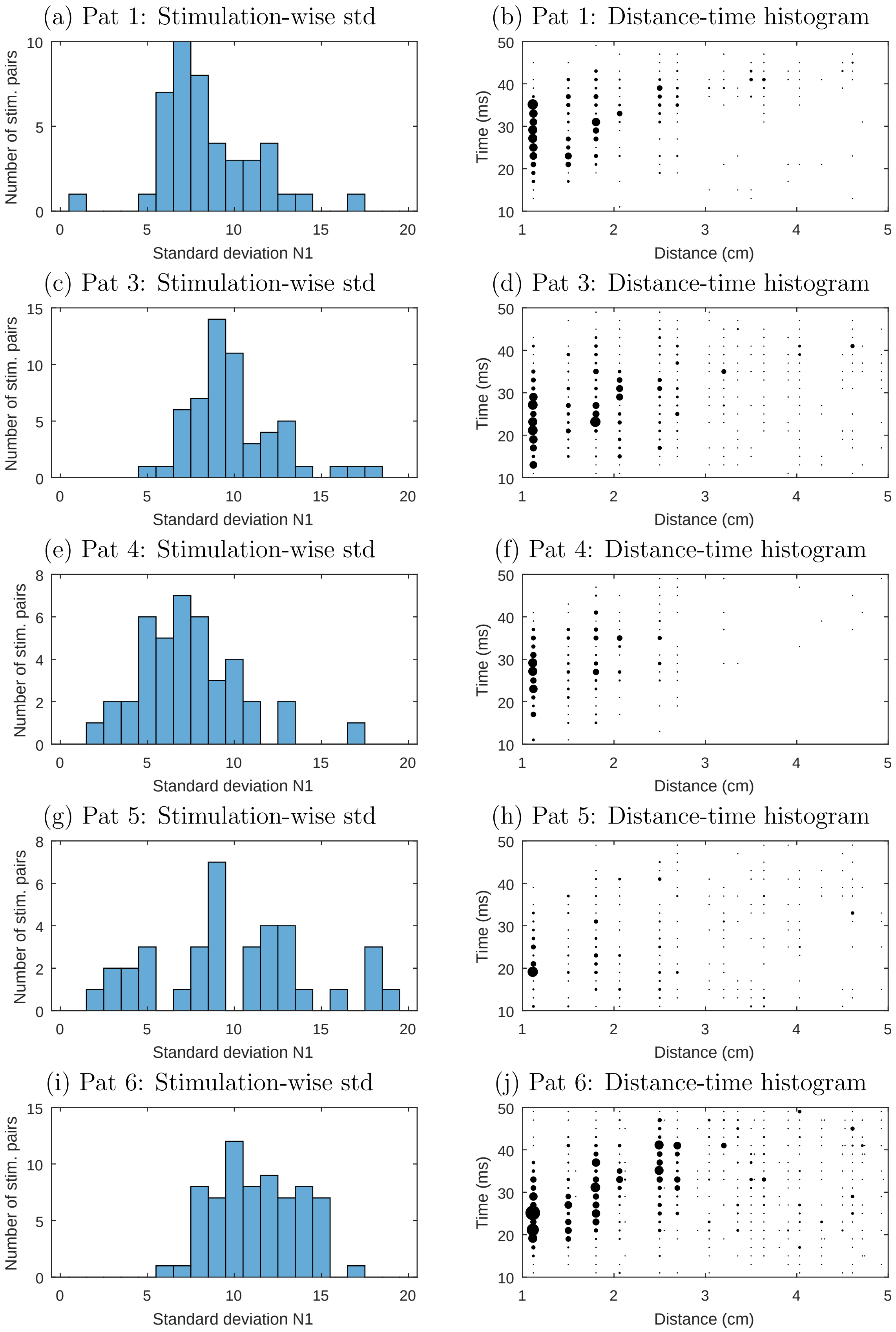

Figure 5: Distribution of the N1 peaks for patients 1, 3-6. (a), (c), (e), (g) and (i) Histogram of the stimulation-wise standard deviation of N1 times. (b), (d), (f), (h) and (j) Histogram showing the distribution of all N1 over both distance to the stimulation pair and peak time. The size of the dots indicate the number of observations.
